# Energy-Efficient Hierarchical Federated Learning in UAV Networks with Partial AI Model Upload Under Non-Convex Loss

**DOI:** 10.3390/s26020619

**Published:** 2026-01-16

**Authors:** Hui Li, Shiyu Wang, Yu Du, Runlei Li, Xin Fan, Chuanwen Luo

**Affiliations:** 1 College of Information Engineering, Taizhou University, Taizhou 225300, China; huili_tzxy@tzu.edu.cn; 2 School of Information Science and Technology, Beijing Forestry University, Beijing 100083, China; wsy2619@bjfu.edu.cn (S.W.); nagasakiianno@bjfu.edu.cn (Y.D.); 3 Hebei Key Laboratory of Smart National Park, Beijing 100083, China; 4 Infrastructure Inspection Research Institute, China Academy of Railway Sciences Corporation Limited, Beijing 100081, China; lirunlei@rails.cn

**Keywords:** Hierarchical Federated Learning, mobility, non-convexity, energy-efficient optimization, partial model upload, Lyapunov optimization

## Abstract

Hierarchical Federated Learning (HFL) alleviates the trade-off between communication overhead and privacy protection in mobile scenarios via multi-level aggregation and mobility consideration. However, its idealized convex loss assumption and full-dimension parameter upload deviate from real-world non-convex tasks and edge channel constraints, causing excessive energy consumption, high communication cost, and compromised convergence that hinder practical deployment. To address these issues in mobile/UAV networks, this paper proposes an energy-efficient optimization scheme for HFL under non-convex loss, integrating a dynamically adjustable partial-dimension model upload mechanism. By screening key update dimensions, the scheme reduces uploaded data volume. We construct a total energy minimization model that incorporates communication/computation energy formulas related to upload dimensions and introduces an attendance rate constraint to guarantee learning performance. Using Lyapunov optimization, the long-term optimization problem is transformed into single-round solvable subproblems, with a step-by-step strategy balancing minimal energy consumption and model accuracy. Simulation results show that compared with the original HFL algorithm, our proposed scheme achieves significant energy reduction while maintaining high test accuracy, verifying the positive impact of mobility on system performance.

## 1. Introduction

The popularization of the Sixth-Generation (6G) communication technology, Artificial Intelligence (AI) and the Internet of Things (IoT) has spawned massive distributed terminal data [1,2,3]. To gain knowledge from these data to support various applications, traditional centralized machine learning struggles to meet the requirements of mobile scenarios due to high communication overhead and significant risks of data privacy leakage [4,5,6]. Conventional Federated Learning (FL) adopts a decentralized paradigm of “local training + model aggregation” [7,8], which effectively reduces the amount of data upload and protects user privacy. Hierarchical Federated Learning (HFL) further introduces edge servers, upgrading the “user–cloud” two-tier architecture to a “user–edge–cloud” three-tier architecture [9]. Clients train local datasets and upload updated models to edge servers for edge aggregation. After several rounds of edge aggregation, edge servers upload the models to the cloud server for global aggregation. Through “many-to-one” parameter compression, long-distance communication overhead is significantly reduced. Since it is the model parameters (rather than users’ raw data) that are uploaded for aggregation, user data privacy is effectively safeguarded [6,10,11].

However, with the exponential growth in the number of terminal devices (e.g., smartphones, drones, etc), an increasing number of deep learning tasks need to be completed in mobile edge scenarios, and the number of model parameters surges with task complexity [6,10,12]. Although HFL can reduce communication energy consumption, challenges persist during model upload. On the one hand, the wireless channel bandwidth of existing mobile terminals is limited, and channel conditions are susceptible to user mobility (e.g., path loss, multipath fading). On the other hand, uploading full-dimensional model parameters leads to increased communication latency and higher data packet loss rates, resulting in training interruptions and wasted resources. In addition, mobile devices have limited battery capacity and computing resources. When the number of model parameters is large, the high communication energy consumption caused by full-dimensional model upload severely shortens device battery life, restricting the practical deployment of HFL especially in mobile computing scenarios such as unmanned aerial vehicle (UAV) networks [13,14,15,16,17].

Existing studies mainly focus on resource allocation or user scheduling but fail to optimize energy consumption from the perspective of model upload dimensions themselves, making it difficult to fundamentally solve the energy consumption problem in mobile scenarios. Accordingly, we propose to upload partial dimension of the model, where each client only uploads key dimension parameters critical to model updates. By considering the differences in the contribution of various dimensions to model updates, additional energy consumption caused by redundant data upload is effectively reduced. This method not only decreases data communication volume but also retains core information through a dimension selection strategy. Building on the architectural advantages of HFL, this innovative scheme further optimizes communication efficiency, preserves the core feature of FL, and addresses the communication and efficiency bottlenecks of traditional architectures, providing a more efficient implementation path for FL training in large-scale distributed scenarios.

Simultaneously, we find that in existing studies, the design of attendance rate mechanisms is mostly based on the assumption of convex loss functions. The core logic is to measure the gradient distance between the user’s local model and the edge/global model, prioritize users with shorter gradient distances to participate in training at high frequencies, and guarantee a basic attendance rate for users with longer gradient distances [18], thereby balancing “improving model aggregation efficiency” and “ensuring data diversity”. Nevertheless, optimization problems in real-world scenarios are more consistent with non-convex characteristics [1,19]. Jiang et al. [20] further point out that existing attendance rate mechanisms with partial worker participation mostly have implicit assumptions of “full participation + convex functions”. However, in practical scenarios with non-convex objective functions and non-IID data, these mechanisms fail to adapt to the distribution of local optima, leading to a more than 40% decrease in convergence speed and even trapping the model in poor local optima [1,20]. Although non-convex loss functions have numerous local optimal solutions, making it difficult to achieve the global optimal solution through conventional gradient descent, existing attendance rate mechanisms under convex assumptions do not fully consider the normality of the prevalent local optima in non-convex scenarios, resulting in significant flaws. This may lead to two types of problems: first, simply attributing users with long gradient distances to large model deviations, excessively restricting their attendance rates while ignoring the unique value of local optimal models of some users under specific data distributions; second, defaulting that models of users with short gradient distances are close to the global optimal solution, without considering that they may be trapped in poor local optima (e.g., local minima with low accuracy), and their high-frequency participation may instead hinder the convergence of the global model. Therefore, there is an urgent need to extend the attendance rate mechanism from convex optimization scenarios to non-convex scenarios, enhancing its adaptability in real-world systems.

Based on the above considerations, we studied energy-efficient HFL in UAV networks with partial model upload under non-convex loss functions. The main contributions of this paper are as follows:

- By integrating the actual hierarchical federated data transmission process, mobility constraints (channel conditions, dwell time), and attendance rate constraints, we design a partial-dimension model upload mechanism. In the process of selecting local model upload and calculating the user attendance rate, we introduce the uploaded model dimension constraint, breaking through the existing Gradient-Based Client Selection Strategy (GBCSS) mechanism that only relies on the single quantitative logic of the gradient divergence, to ensure that the model can both reflect its contribution to aggregation and embody the rationality of upload cost.
- Existing studies have verified the optimization value of non-full-dimension upload for communication energy consumption in Federated Learning (The FedDisco algorithm proposed by Li et al. [21] screens key scalar information of the model through zeroth-order optimization, reducing the communication cost from O(d) (proportional to the model dimension *d*) to a constant O(1), and still achieves convergence accuracy comparable to full-dimension upload in non-convex scenarios). This paper further designs a partial-dimension model upload mechanism for the three-tier architecture of HFL: by dynamically adjusting the uploaded data volume $S_{i}$ of user *i* ($0<S_{i}<S_{\mathrm{full}}$), redundant parameters with small contributions to the model are discarded, and core parameters with large contributions are retained, thereby reducing communication energy consumption from the source. We construct an energy consumption optimization model involving $S_{i}$ by deriving communication/computation energy consumption formulas that include $S_{i}$.
- Aiming at the limitation that traditional HFL only considers the ideal case of convex optimization, we extend it to non-convex scenarios by considering that the model may fall into local optima during gradient descent, which is consistent with the general situation in reality where global optima are often not obtained during model training.
- For the proposed target optimization problem, we design a step-by-step solution strategy. We decompose global energy consumption optimization into three stages: (1) single-user resource allocation [22], (2) low-energy user selection considering gradient differences, and (3) global energy consumption aggregation. Further, the efficient solution is achieved through Lyapunov optimization and the Alternating Direction Method of Multipliers (ADMM) algorithm.

Existing Hierarchical Federated Learning (HFL) studies can be categorized into three types: convex loss assumption-based, full-dimensional upload-based, and static attendance rate-based, with core limitations being detachment from non-convex reality, excessive communication energy consumption, and limited convergence performance, respectively. For non-convex scenarios, this paper integrates dynamic partial-dimension upload and dynamic attendance rate mechanisms, filling the collaborative gap of “low energy consumption—dynamic optimization—performance guarantee” for HFL in non-convex scenarios.

The rest of this paper is organized as follows: In Section 2, the system model is described with problem definitions. In Section 3, the proposed algorithm is provided and then tested in Section 4. Finally, Section 5 presents the summary of the paper.

## 2. System Model

**Figure 1:**
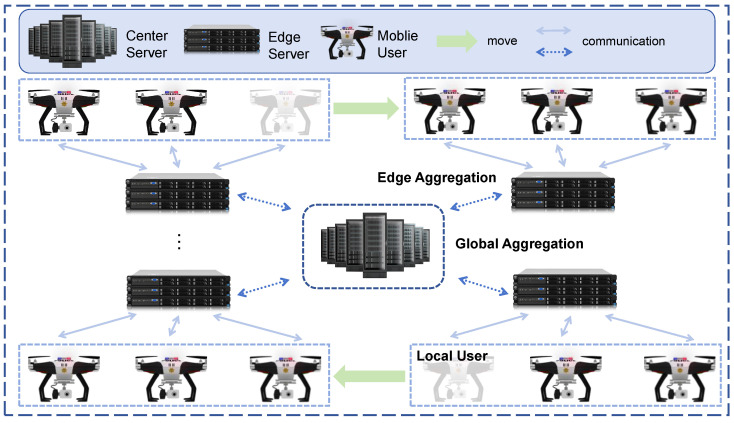
HFL system model.

HFL adopts a three-tier architecture of “central server–edge server–mobile user” [23] to implement distributed model training, workflow of which is illustrated in Figure 1. In the local update phase, mobile users complete the preliminary training of model parameters based on their local data. After local training is finished, mobile users upload the model parameters to their affiliated edge servers. The edge servers perform regional-level aggregation on the model parameters of users within their jurisdiction to obtain edge-layer models [24]. Subsequently, the edge servers upload the aggregated model parameters to the central server. After the central server completes global model aggregation, it distributes the updated global model to each edge server. Finally, each edge server synchronizes the model parameters to the mobile users under its jurisdiction, completing one training iteration [25]. This hierarchical architecture effectively reduces the communication load of the central server through localized aggregation at the edge layer, while ensuring the distributed nature of model training.

**Figure 2:**
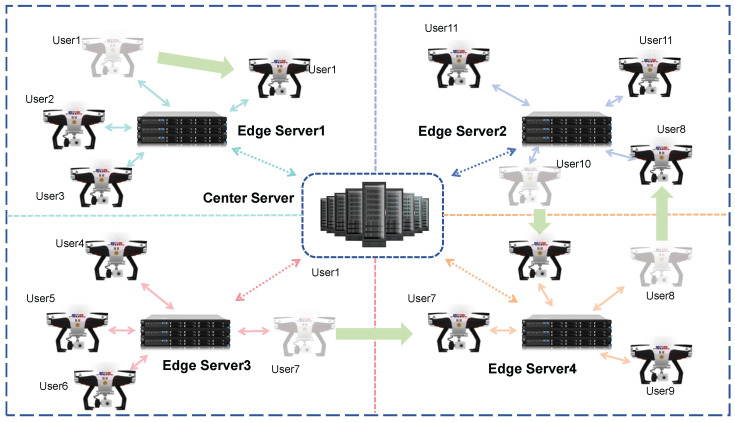
Mobility model for HFL.

In practical application scenarios, HFL needs to address multiple challenges caused by dynamic topology changes of mobile users, as shown in Figure 2. Users (e.g., User1, User7, User11) exhibit scenarios such as cross-region movement (e.g., User1 migrates from the coverage area of Edge Server1 to other edge service areas) and irregular movement within a region. First, the issue of communication energy consumption and efficiency is prominent: if mobile users upload full-dimensional model parameters in areas with poor channel quality, it is prone to packet loss and significant increase in transmission delay due to channel fading, and even the training process may be interrupted because the transmission of full-dimensional parameters takes too long [24]. Second, the complexity of user selection and resource scheduling increases significantly. After users move across regions, they need to re-access new edge servers. If the matching strategy between users and edge servers is not optimized in a timely manner, the system energy consumption will remain high due to channel resource competition and redundant transmission of model parameters [26]. In addition, the irregular movement of mobile users will further exacerbate the non-independent and identically distributed (non-IID) characteristics of data distribution [27]. Without an adaptive model parameter fragmentation and upload strategy (e.g., dynamic switching between full-dimensional and key-dimensional uploads) [28], it will be difficult to achieve an effective balance between model accuracy and communication overhead, ultimately restricting the performance and practical deployment value of HFL.

### 2.1. Derivation of Global Total Energy Consumption

When considering the derivation of energy consumption issues in the novel HFL, it is necessary to dynamically adjust the dimension of the uploaded AI model $S_{i}$ at the *i*-th user (where $0<S_{i}<S_{\mathrm{full}}$, $S_{\mathrm{full}}$ denotes the full dimension of the AI model) under the premise of ensuring validation accuracy, so as to reduce communication energy consumption from the source. Define the model validation accuracy as ρ, and the relationship between ρ and $S_{i}$ is expressed as [22,29]

(1) $$\rho =aS_{i}^{-b},$$

where the value of the error rate ρ can be determined arbitrarily according to the desired model performance, and *a* and *b* are empirical parameters that need to be fitted from data. The quantitative form of this power-law relationship refers to the research results in [22,29], which verified the correlation between model size and recognition accuracy through experiments, providing a basis for the accuracy–energy consumption balance design of $S_{i}$ in this paper. Then, the rate of change between model size and recognition accuracy is given by

(2) $$\frac{d\rho }{dS}=\rho^{\prime }=-abS_{i}^{-b-1}$$

From this, two constraint conditions for $S_{\min}$ are derived as follows: When ρ is user-defined as $\rho_{\max}$ (the maximum value of ρ), we have

(3) $$S_{i}={\left(\frac{a}{\rho_{\max}}\right)}^{\frac{1}{b}}.$$

Under the trade-off between recognition accuracy (Acc) and energy consumption, we have

(4) Acc=1−ρ,

and

(5) $$\frac{d(\mathrm{Acc})}{dS_{i}}={\left(1-\rho \right)}^{\prime }=abS_{i}^{-b-1}.$$

As the model size $S_{i}$ continues to increase, the recognition accuracy (Acc) gradually converges. Here, we define Acc in (4) as “benefit” and the energy consumption *E* as “cost”, and then consider the functional relationship between “cost” and “benefit” as follows. Let the bandwidth of a Resource Block (RB) be denoted as *C*. We assume that each selected user is assigned to one channel. According to the Shannon capacity formula [30,31,32], the uplink data transmission rate of the *i*-th user in the *n*-th round is given by

(6) $$r_{i,n}^{\mathrm{up}}=C\log_{2}\left(1+\frac{p_{i}^{n}{\left(h_{i}^{n}\right)}^{2}}{N_{0}C}\right),$$

where $p_{i,n}$ is the transmit power of the *i*-th user, and $h_{i,n}$ is the uplink channel response of the *i*-th user, which follows a Rayleigh distribution with path loss. The path loss model is given by $35.0+35.0\log_{10}D\;\mathrm{dB}$ [5], where *D* is the distance between the user and the Base Station (BS) in kilometers (km), and $N_{0}$ is the noise power spectral density. Considering that the power and bandwidth of the BS are sufficient, the download delay is negligible compared with the total delay. Therefore, we focus mainly on uplink transmission. Given the uploaded model size $S_{i}$, the upload delay is given by

(7) $$T_{i,n}^{\mathrm{transmit}}=\frac{S_{i}}{r_{i,n}^{\mathrm{up}}}.$$

Then the upload energy consumption is given by

(8) $$E_{i,n}^{\mathrm{transmit}}=p_{i}^{n}T_{i,n}^{\mathrm{transmit}}=\frac{p_{i}^{n}S_{i}}{C\log_{2}\left(1+\frac{p_{i}^{n}{\left(h_{i}^{n}\right)}^{2}}{N_{0}C}\right)}.$$

The local update computation delay is given by

(9) $$T_{i,n}^{\mathrm{calculate}}=\frac{\gamma \tau_{l}\left|D_{i}\right|}{g_{i}^{n}},$$

where γ is the time period for the CPU to compute one sample, $g_{i}^{n}$ is the computation frequency, $\tau_{l}$ is the local epoch number, and $|D_{i}|$ represents the data size of dataset $D_{i}$. The computation energy consumption is obtained as

(10) $$E_{i,n}^{\mathrm{calculate}}=\alpha \tau_{l}\gamma \left|D_{i}\right|{\left(g_{i}^{n}\right)}^{2}=\frac{\alpha \tau_{l}^{3}\gamma^{3}{\left|Di\right|}^{3}}{{\left(T_{i,n}^{\mathrm{transmit}}\right)}^{2}},$$

where α is coefficient for the energy consumption.

From this, the functional relationship between the total energy consumption (i.e., “cost”) and the model size $S_{i}$ is, respectively, derived as

(11) $$E_{\mathrm{total}}=E_{i,n}^{\mathrm{transmit}}+E_{i,n}^{\mathrm{calculate}},$$

where

(12) $$\frac{dE_{\mathrm{total}}}{dS_{i}}=C_{1}+C_{2}S_{i}^{-3}.$$

where $C_{1}=\frac{p_{i}^{n}}{C\log_{2}\left(1+\frac{p_{i}^{n}{\left(h_{i}^{n}\right)}^{2}}{N_{0}C}\right)}$ and $C_{2}=-2\alpha \tau_{l}^{3}\gamma^{3}{\left|D_{i}\right|}^{3}{\left(r_{i,n}^{\mathrm{up}}\right)}^{2}$.

To achieve lower energy consumption while ensuring accuracy and to meet the goal of positive profit, it is necessary to ensure that the marginal growth rate of the energy consumption “cost” is less than that of the accuracy “benefit”. This constraint can be formally expressed as $\frac{dE_{\mathrm{total}}}{dS_{i}}<\frac{d(\mathrm{Acc})}{dS_{i}}$, leading to

(13) $$abS_{i}^{-b-1}\geq C_{1}+C_{2}\cdot S_{i}^{-3}.$$

The Newton–Raphson method [33] is used to solve the above derivation (13) numerically for two cases: (1) the special case b=2 and (2) the general case b≠2, which are given as follows:

(14) $$\left\{\begin{array}{cc} b=2, & S_{i}\leq \sqrt[{3}]{\frac{ab-C_{2}}{C_{1}}}\;\left(C_{1}\neq 0,ab>C_{2}\right),\\ b\neq 2, & S_{i}=S_{i}^{n+1}, \end{array}\right.$$

where $S_{i}^{n+1}$ is the result at the (n+1)-th iteration of the Newton–Raphson method.

As a result, the constraint on model size is derived as

(15) $$S_{i}\geq S_{\min}=\max\left(S_{1},S_{2}\right),$$

where $S_{i,1}={\left(\frac{a}{\rho_{\max}}\right)}^{\frac{1}{b}}$ and $S_{i,2}=\left\{\begin{array}{cc} \sqrt[{3}]{\frac{ab-C_{2}}{C_{1}}}, & b=2\\ S_{i}^{n+1}, & b\neq 2. \end{array}\right.$

Focusing on the overall goal of reducing energy consumption, we have restricted the size of the uploaded model $S_{i}$. To further optimize HFL, the following optimization problem is proposed as

(16a) $$\begin{array}{cc} P1:\; & \min_{a,p,g,S_{i}}\sum_{n=1}^{N}\sum_{i\in B}a_{i}^{n}\left(E_{i,n}^{\mathrm{transmit}}+E_{i,n}^{\mathrm{calculate}}\right) \end{array}$$

(16b) $$\begin{array}{cc} s.t.\; & C1:a_{i}^{n}\in \left\{0,1\right\}, \end{array}$$

(16c) $$\begin{array}{cc} & C2:\sum_{i\in C_{e}^{n}}a_{i}^{n}\leq M_{e}, \end{array}$$

(16d) $$\begin{array}{cc} & C3:a_{i}^{n}\left(T_{i,n}^{\mathrm{transmit}}+T_{i,n}^{\mathrm{calculate}}\right)\leq T_{i,n}^{\max}, \end{array}$$

(16e) $$\begin{array}{cc} & C4:g_{i}^{\min}\leq g_{i}^{n}\leq g_{i}^{\max}, \end{array}$$

(16f) $$\begin{array}{cc} & C5:0\leq p_{i}^{n}\leq p_{i}^{\max}, \end{array}$$

(16g) $$\begin{array}{cc} & C6:\frac{1}{N}\sum_{n=1}^{N}a_{i}^{n}\geq \frac{1}{N}\sum_{n=1}^{N}\Gamma_{i}^{n}, \end{array}$$

(16h) $$\begin{array}{cc} & C7:S_{\min}\leq S_{i}\leq S_{\mathrm{full}}. \end{array}$$

Table 1 summarizes the key notations.

### 2.2. Gradient-Based Client Selection Strategy

In the original HFL framework, after completing local iterative training, users are required to upload their full-dimensional local model parameters $w_{i}^{\tau_{l},n}$ to edge servers. Edge servers aggregate the full-dimensional parameters of all users to obtain the edge model $v_{e}^{n}$, which is further uploaded to the central server for global model update. Although this full-dimensional parameter upload mechanism can ensure the integrity of model aggregation, it has limitations such as high communication overhead, significant privacy leakage risk, and heavy computational pressure on edge devices. In this paper, through the strategy of selectively uploading partial model dimensions $S_{i}^{\prime \prime }$, we achieve multi-dimensional optimization of communication overhead, privacy protection, and computational efficiency while retaining the advantages of the HFL architecture.

In this subsection, a Gradient-Based Client Selection Strategy (GBCSS) (also referred to as attendance rate selection in this paper) is proposed via model divergence constraints to determine whether the model will be selected to participate in aggregation.

We evaluate the learning performance of user *i* based on the divergence between the local model $w_{i}^{\tau_{l},n}$ and the auxiliary edge model $v_{e}^{\tau ,n}$ to decide whether to select this local model. The auxiliary edge model represents the auxiliary edge model parameters of edge server *e* after the τ-th virtual iteration in the *n*-th communication round. Essentially, it is a “centralized ideal model” constructed by the edge server based on the dataset $C_{e}^{n}$ of users within its coverage under the scenario of non-federated centralized training. The auxiliary edge model parameters $v_{e}^{\tau ,n}$ follow centralized gradient descent with the dataset $C_{e}^{n}$. It will be synchronized with the edge model $w_{e}^{n}$ after edge aggregation. Therefore, the update method of $v_{e}^{\tau ,n}$ is as follows:

(17) $$\begin{array}{c} v_{e}^{\tau ,n}=\left\{\begin{array}{cc} w_{e}^{n}, & \tau =0\\ v_{e}^{\tau -1,n}-\eta \nabla F_{e}^{n}\left(v_{e}^{\tau -1,n}\right), & \tau \neq 0 \end{array}\right., \end{array}$$

where $F_{e}^{n}\left(\cdot \right)$ is the loss function at the edge server.

To conduct quantitative analysis, we make the following assumptions about the loss functions commonly used in FL analysis.

**Assumption** **A1.** *The loss function of the i-th user $f_{i}\left(w\right)$ is non-convex.*

**Assumption** **A2.** *$f_{i}\left(w\right)$ is ρ-Lipschitz, i.e.,*

(18) $$\begin{array}{c} ∥f_{i}\left(w_{1}\right)-f_{i}\left(w_{2}\right)∥\leq \rho ∥w_{1}-w_{2}∥,\;\forall w_{1},w_{2}. \end{array}$$

**Assumption** **A3.** *$f_{i}\left(w\right)$ is β-smooth, i.e.,*

(19) $$\begin{array}{c} ∥\nabla f_{i}\left(w_{1}\right)-\nabla f_{i}\left(w_{2}\right)∥\leq \beta ∥w_{1}-w_{2}∥,\;\forall w_{1},w_{2}. \end{array}$$

**Assumption** **A4.** *There exists an upper bound on the distance between the gradient of the local loss function and that of the edge loss function, i.e.,*

(20) $$\begin{array}{c} ∥\nabla f_{i}\left(w\right)-\nabla F_{e}^{n}\left(w\right)∥\leq \delta_{i}^{n}\cdot \frac{S_{i}}{S_{\mathrm{full}}},\;i\in C_{e}^{n}. \end{array}$$

**Assumption** **A5.** *There exists an upper bound on the distance between the gradient of the edge loss function and that of the global loss function, i.e.,*

(21) $$\begin{array}{c} ∥\nabla F_{e}^{n}\left(w\right)-\nabla F\left(w\right)∥\leq \Delta_{e}^{n}. \end{array}$$

Regarding the problem proposed in Assumption 1, where the distance between the gradient of the local loss function and that of the edge loss function under non-convex conditions, as well as the distance between the gradient of the edge loss function and that of the global loss function, both have upper bounds. We find that these hold regardless of convexity or non-convexity when the parameter dimension is finite. The derivation is presented as follows.

The loss function $F_{e}^{n}\left(w\right)$ of edge server *e* is a weighted average of the local losses of the users it covers, where the weight is determined by the dataset size $|D_{i}|$ (the larger the data volume, the higher the contribution to the edge loss). Thus, we have

(22) $$\begin{array}{c} F_{e}^{n}\left(w\right)=\frac{\sum_{i\in C_{e}^{n}}\left|D_{i}\right|f_{i}\left(w\right)}{\sum_{i\in C_{e}^{n}}\left|D_{i}\right|}. \end{array}$$

Its gradient is a weighted average of local gradients as follows:

(23) $$\begin{array}{c} \nabla F_{e}^{n}\left(w\right)=\frac{\sum_{i\in C_{e}^{n}}\left|D_{i}\right|\nabla f_{i}\left(w\right)}{\sum_{i\in C_{e}^{n}}\left|D_{i}\right|}. \end{array}$$

We apply the triangle inequality and β-smoothness to $\delta_{i}^{n}$ as

(24) $$\begin{array}{c} \delta_{i}^{n}=∥\nabla f_{i}\left(w\right)-\nabla F_{e}^{n}\left(w\right)∥=∥\nabla f_{i}\left(w\right)-\sum_{k\in C_{e}^{n}}q_{k}^{n}\nabla f_{k}\left(w\right)∥. \end{array}$$

Expanding this, we obtain

(25) $$\begin{array}{c} \delta_{i}^{n}\leq \sum_{k\in C_{e}^{n}}q_{k}^{n}∥\nabla f_{i}\left(w\right)-\nabla f_{k}\left(w\right)∥\leq \sum_{k\in C_{e}^{n}}q_{k}^{n}\cdot \beta ∥w_{i}-w_{k}∥, \end{array}$$

where $∥w_{i}-w_{k}∥$ is the model parameter difference between user *i* and other user *k* within the edge server. Due to $S_{i}<S_{\mathrm{full}}$ and controllable local training parameter values, $∥w_{i}-w_{k}∥$ has an upper bound (denoted as $W_{\max}$). Therefore, $\delta_{i}^{n}\leq \beta W_{\max}$ (the upper bound is determined by β and the maximum parameter difference). Similarly, the edge loss function and the global loss function also have an upper bound $\Delta_{e}^{n}\leq \beta W_{\max}$. Thus, in the non-convex scenario, the optimized design under the non-convex assumption and $S_{\min}<S_{i}<S_{\mathrm{full}}$ not only does not negate the upper bound of the gradient distance but also brings the theoretical basis of the upper bound closer to the non-convex situation in real-world scenarios, which is more in line with the actual training characteristics of deep models (e.g., CNN, where loss functions have local optima). The preservation of Lipschitz β-smoothness ensures the controllability of gradient changes. The range constraint of $S_{i}$ avoids the gradient redundancy of full-dimensional model upload or the insufficient gradient information of extremely few-dimensional model upload, limiting parameter differences and gradient scales to more controllable intervals. Ultimately, the upper bounds of $\delta_{i}^{n}$ and $\Delta_{e}^{n}$ are easier to calculate, and non-convexity does not break the boundedness of gradient distances. The range constraint of $S_{i}$ also enhances the rationality of boundedness.

Meanwhile, in the upper bound constraint of Assumption 4, we incorporate the optimization of model dimension $S_{i}$ by using a differentiated constraint design to make the upper bound of the local loss function and the edge loss function converge to $\beta W_{\max}\cdot \frac{S_{i}}{S_{\mathrm{full}}}$, enabling model dimensions of different scales to participate effectively. Users with larger model dimensions upload more complete information and have a higher tolerance for gradient differences. Users with smaller dimensions upload less information and require stricter gradient alignment.

Based on these assumptions, we can easily derive Lemma 1 and Theorem 1 as follows.

**Lemma** **1.** *$F_{e}^{n}\left(w\right)$ and F(w) are non-convex, ρ-Lipschitz, and β-smooth.*

**Theorem** **1.** *If Assumptions 1, 3 and 4 hold and $i\in C_{e}^{n}$, then*

(26) $$\begin{array}{c} ∥w_{i}^{\tau ,n}-v_{e}^{\tau ,n}∥\leq \theta_{i}^{n}=W_{\max}\cdot \frac{S_{i}}{S_{\mathrm{full}}}\left({\left(1+\eta \beta \right)}^{\tau }-1\right). \end{array}$$

The proofs are so easy, and hence omitted here. According to Theorem 1, we observe that a smaller $W_{\max}$ leads to a smaller $\delta_{i}^{n}$, which is consistent with the trend of traditional FL. When the data distribution of local users is more consistent with that of the edge server, the user’s contribution to FL performance is more significant. The GBCSS $\Gamma_{i}^{n}$ is defined as follows:

(27) $$\begin{array}{c} \Gamma_{i}^{n}=\min\left\{M_{e}\frac{\frac{1}{\theta_{i}^{n}}}{\sum_{i\in C_{e}^{n}}\frac{1}{\theta_{i}^{n}}},1\right\}. \end{array}$$

Considering that the user set of an edge server may change over time, leading to time-varying gradient dispersion, we average GBCSS, as specified in Constraint C6.

## 3. Hierarchical FEDNC-DU Algorithm (HFDA)

The overall objective P1 proposed above is a stochastic optimization problem under the global constraint C6. However, since user responses and channel responses cannot be predicted in advance, it is necessary to optimize this long-term problem into a short-term per-round problem. Here, we adopt the Lyapunov method and solve it using an online algorithm. A virtual debt queue $Q_{i}$ is introduced for each user as a reference factor for scheduling this user. In this way, the debt queue length $Q_{i}\left(n\right)$ is directly related to the user’s GBCSS deviation and follows the dynamic update rule as follows:

(28) $$Q_{i}\left(n+1\right)=\max\left\{Q_{i}\left(n\right)-a_{i}^{n}+\Gamma_{i}^{n},0\right\}.$$

Then, the time-average inequality constraint C6 in the global state is converted into the per-round stability constraint ${C6}^{\prime }$ for the queue.

(29) $$\lim_{n\to \infty }\frac{E\{|Q_{i}\left(n\right)|\}}{n}=0.$$

Define the Lyapunov function L(n) as

(30) $$L\left(n\right)=\frac{1}{2}\sum_{i\in U}Q_{i}{\left(n\right)}^{2}$$

Then, we can derive the Lyapunov drift function ΔL(n) as

(31) ΔL(n)=E{L(n+1)−L(n)∣Q(n)}

Further, construct the Lyapunov drift-plus-penalty function as

(32) $$\Delta_{V}^{n}=V\sum_{i\in U}\left(VE_{i,n}^{\mathrm{transmit}}+VE_{i,n}^{\mathrm{calculate}}\right)+\Delta L\left(n\right),$$

where $V\sum (E_{i,n}^{\mathrm{transmit}}+E_{i,n}^{\mathrm{calculate}})$ is the penalty term, ΔL(n) is the drift term, and parameter *V* is the trade-off control parameter in the Lyapunov optimization framework, balancing energy consumption and long-term participation rate constraints.

When minimizing ΔL(n), it can ensure that the time-average debt $\frac{E\{|Q_{i}\left(n\right)|\}}{n}$ of $Q_{i}\left(n\right)$ approaches 0 as *n* increases, i.e., satisfying constraint C6. Therefore, for any debt queue $Q_{i}\left(n\right)$, ΔL(n) is bounded by

(33) $$\Delta L\left(n\right)\leq U+\sum_{i\in U}Q_{i}\left(n\right)\left(\Gamma_{i}^{n}-a_{i}^{n}\right).$$

It can be derived that

(34) $$\Delta_{V}^{n}\leq \sum_{i\in U}a_{i}^{n}\left(VE_{i,n}^{\mathrm{transmit}}+VE_{i,n}^{\mathrm{calculate}}-Q_{i}\left(n\right)\right)+C.$$

To reduce total energy consumption while ensuring long-term participation rate constraints, we can minimize the Lyapunov drift-plus-penalty function $\Delta_{V}^{n}$. Thus, Problem P2 is obtained as

(35a) $$\begin{array}{cc} \min_{a,p,g} & \sum_{i\in U}a_{i}^{n}\left(VE_{i,n}^{\mathrm{transmit}}+VE_{i,n}^{\mathrm{calculate}}-Q_{i}\left(n\right)\right) \end{array}$$

(35b) $$\begin{array}{cc} s.t. & \;C1∼C5 \end{array}$$

The analysis of transmission energy consumption leads to the following findings:

(36) $$\frac{\partial^{2}E_{i,n}^{\mathrm{transmit}}}{{\left(\partial T_{i,n}^{\mathrm{transmit}}\right)}^{2}}=\frac{N_{0}S_{i}^{2}{\left(\ln2\right)}^{2}}{{\left(h_{i}^{n}\right)}^{2}B^{2}{\left(T_{i,n}^{\mathrm{transmit}}\right)}^{3}2^{\frac{S_{i}}{BT_{i,n}^{\mathrm{transmit}}}}}>0.$$

(37) $${\left.\frac{\partial E_{i,n}^{\mathrm{transmit}}}{\partial T_{i,n}^{\mathrm{transmit}}}\right|}_{T_{i,n}^{\mathrm{transmit}}\to +\infty }=0.$$

Thus, we have $\frac{\partial E_{i,n}^{\mathrm{calculate}}}{\partial T_{i,n}^{\mathrm{calculate}}}<0$, i.e.,

(38) $$\frac{\partial E_{i,n}^{\mathrm{calculate}}}{\partial T_{i,n}^{\mathrm{calculate}}}=-\frac{2\alpha \tau_{l}^{3}\gamma^{3}{\left|D_{i}\right|}^{3}}{{\left(T_{i,n}^{\mathrm{calculate}}\right)}^{3}}<0.$$

Thus, the time *T* is a key variable. Using more time can reduce energy consumption. We replace the computation frequency *f* and transmission power *p* with communication time *t*, leading to Problem P3 as

(39a) $$\begin{array}{cc} P3:\; & \min_{a,t}\sum_{i\in U}a_{i}^{n}R_{i}^{n}\left(t_{i}^{n}\right) \end{array}$$

(39b) $$\begin{array}{cc} s.t.\; & C1,C2, \end{array}$$

(39c) $$\begin{array}{cc} & C4^{\prime }:g_{i}^{\min}\leq \frac{\gamma \tau_{l}\left|D_{i}\right|}{T_{i,n}^{\max}-t_{i}^{n}}\leq g_{i}^{\max}, \end{array}$$

(39d) $$\begin{array}{cc} & C5^{\prime }:0\leq \frac{N_{0}\left(2^{\frac{S_{i}}{Bt_{i}^{n}}}-1\right)}{{\left(h_{i}^{n}\right)}^{2}}\leq P_{i}^{\max}. \end{array}$$

### 3.1. Single-User Optimization

The current problem is transformed into P3 to minimize $\sum_{i\in U}a_{i}^{n}R_{i}^{n}\left(t_{i}^{n}\right)$. P3 is a mixed-integer non-convex optimization problem, which is complex to solve. Thus, it is further decomposed into a user selection subproblem and a time allocation subproblem. Therefore, for each selected user *i*, the goal is to minimize $R_{i}^{n}\left(t_{i}^{n}\right)$, leading to Problem P4

(40) $$\begin{array}{cc} P4:\; & \min_{t_{i}^{n}}R_{i}^{n}\left(t_{i}^{n}\right)\\ s.t.\; & C4^{\prime },C5^{\prime }. \end{array}$$

For the function $R_{i}^{n}\left(t_{i}^{n}\right)$, we have

(41) $$\frac{\partial^{2}R_{i}^{n}\left(t_{i}^{n}\right)}{\partial {\left(t_{i}^{n}\right)}^{2}}=\frac{6V\alpha \tau_{l}^{3}\gamma^{3}{\left|D_{i}\right|}^{3}}{{\left(T_{i,n}^{\max}-t_{i}^{n}\right)}^{4}}+\frac{VN_{0}S_{i}^{2}{\left(\ln2\right)}^{2}}{{\left(h_{i}^{n}\right)}^{2}B^{2}{\left(t_{i}^{n}\right)}^{3}2^{\frac{S_{i}}{Bt_{i}^{n}}}}>0.$$

The second-order derivative is strictly greater than 0, indicating that $R_{i}^{n}\left(t_{i}^{n}\right)$ is a strictly convex function.

For the given constraints ${C4}^{\prime }$ and ${C5}^{\prime }$, the solution is derived as

(42) $$T_{i,n}^{\max}-\frac{\gamma \tau_{l}\left|D_{i}\right|}{g_{i}^{\min}}\leq t_{i}^{n}\leq T_{i,n}^{\max}-\frac{\gamma \tau_{l}\left|D_{i}\right|}{g_{i}^{\max}},$$

and

(43) $$t_{i}^{n}\geq \frac{S_{i}}{B\log_{2}\left(1+\frac{P_{i}^{\max}{\left(h_{i}^{n}\right)}^{2}}{N_{0}}\right)},$$

which follows that

(44) $$t_{i}^{\mathrm{left}}=\max\left\{T_{i,n}^{\max}-\frac{\gamma \tau_{l}\left|D_{i}\right|}{g_{i}^{\min}},\frac{S_{i}}{B\log_{2}\left(1+\frac{{\left(h_{i}^{n}\right)}^{2}P_{i}^{\max}}{N_{0}}\right)}\right\},$$

and

(45) $$t_{i}^{\mathrm{right}}=T_{i,n}^{\max}-\frac{\gamma \tau_{l}\left|D_{i}\right|}{g_{i}^{\max}},$$

where $t_{i}^{\mathrm{left}}$ represents that the minimum communication time satisfying constraints (derived from the transmit power upper bound in ${C5}^{\prime }$ to ensure power does not exceed $P_{i}^{\max}$), and $t_{i}^{\mathrm{right}}$ represents that the maximum communication time satisfying constraints (derived from the computation frequency lower bound in ${C4}^{\prime }$ to ensure computation frequency is no less than $g_{i}^{\min}$).

Considering that $R_{i}^{n}\left(t_{i}^{n}\right)$ is a convex function, the optimal solution occurs at endpoints or extreme points, leading to the following three cases.

**Case 1: Non-existent Constraint Interval (User Not Selectable)**: If $t_{i}^{\mathrm{left}}>t_{i}^{\mathrm{right}}$, it indicates the time interval satisfying all constraints (C4’, C5’) is empty. The user cannot complete model upload under hardware/latency constraints, so the user is not selected, and its cost is set to +∞ (denoting infeasibility).

**Case 2: Existing Constraint Interval and Monotonic Function (Optimal Solution at Boundary)**: If $t_{i}^{\mathrm{left}}\leq t_{i}^{\mathrm{right}}$, compute the first-order derivatives (gradients) $\nabla R_{i}^{n}\left(t_{i}^{\mathrm{left}}\right)$ and $\nabla R_{i}^{n}\left(t_{i}^{\mathrm{right}}\right)$ at interval endpoints $t_{i}^{\mathrm{left}}$ and $t_{i}^{\mathrm{right}}$. If  $\nabla R_{i}^{n}\left(t_{i}^{\mathrm{left}}\right)\cdot \nabla R_{i}^{n}\left(t_{i}^{\mathrm{right}}\right)\geq 0$, the function is monotonic (with unchanged gradient sign) over $\left[t_{i}^{\mathrm{left}},t_{i}^{\mathrm{right}}\right]$. Thus, the optimal solution lies at the interval endpoints, and we take the time corresponding to $\min\{R_{i}^{n}\left(t_{i}^{\mathrm{left}}\right),R_{i}^{n}\left(t_{i}^{\mathrm{right}}\right)\}$ as $t_{i}^{n}$.

**Case 3: Existing Constraint Interval and Non-monotonic Function (Optimal Solution Inside, Solved by Newton’s Method)**: If $t_{i}^{\mathrm{left}}\leq t_{i}^{\mathrm{right}}$ and $\nabla R_{i}^{n}\left(t_{i}^{\mathrm{left}}\right)\cdot \nabla R_{i}^{n}\left(t_{i}^{\mathrm{right}}\right)<0$, the function changes from increasing to decreasing (or vice versa) within the interval, and there exists an interior point where the gradient is zero, i.e.,

(46) $$\nabla R_{i}^{n}\left(t_{i}^{n*}\right)=0.$$

We solve for this interior point using Newton’s iterative method, with detailed steps provided in Algorithm 1.
**Algorithm 1** Solution of P4.1:**Input:**2:$\;T_{i,n}^{\max},\gamma ,\tau_{l},,g_{i}^{\min},g_{i}^{\max},V,N_{0},C,h_{i}^{n},Q_{i}\left(n\right),\sigma ,r.$3:Calculate $t_{i}^{\mathrm{left}},t_{i}^{\mathrm{right}},\nabla R_{i}^{n}\left(t_{i}^{\mathrm{left}}\right),\nabla R_{i}^{n}\left(t_{i}^{\mathrm{right}}\right)$4:**if** $t_{i}^{\mathrm{left}}>t_{i}^{\mathrm{right}}$ **then**5:   set $t_{i}^{n,*}=\mathrm{NULL}$ and $R_{i}^{n}\left(t_{i}^{n,*}\right)=+\infty$.6:**else if** $\nabla R_{i}^{n}\left(t_{i}^{\mathrm{left}}\right)\nabla R_{i}^{n}\left(t_{i}^{\mathrm{right}}\right)\geq 0$ **then**7:   $R\left(t_{i}^{n,*}\right)=\min\left\{R_{i}^{n}\left(t_{i}^{\mathrm{left}}\right),R_{i}^{n}\left(t_{i}^{\mathrm{right}}\right)\right\},$8:   $t_{i}^{n,*}=\arg\min_{t}\left\{R_{i}^{n}\left(t_{i}^{\mathrm{left}}\right),R_{i}^{n}\left(t_{i}^{\mathrm{right}}\right)\right\}.$9:**else**10:   $iter=0,\;t=t_{i}^{\mathrm{left}}.$11:   **while** $|\nabla R_{i}^{n}\left(t\right)|>\sigma$ and iter<r **do**12:     $t=t-\nabla^{2}R_{i}^{n}{\left(t\right)}^{-1}\nabla R_{i}^{n}\left(t\right).$13:     iter=iter+1.14:   **end while**15:   $t_{i}^{n,*}=t.$16:**end if**17:**Output:** $t_{i}^{n,*},R_{i}^{n}\left(t_{i}^{n,*}\right)$

### 3.2. Multi-User Selection

Based on the above, we have derived the minimization of $R_{i}^{n}\left(t_{i}^{n}\right)$ for each user *i*. Now, the problem lies in precisely selecting low-cost users, leading to Problem P5 as follows:

(47) $$P5:\min_{a}\sum_{i\in U}a_{i}^{n}R_{i}^{n}\left(t_{i}^{n,*}\right)\;s.t.\;C1,C2.$$

The optimal cost $R_{i}^{n}\left(t_{i}^{n,*}\right)$ of each user is determined solely by its own channel, data, and hardware parameters, independent of the selection of other users.

Based on this characteristic, the greedy algorithm, which selects the user with the lowest cost each time, can efficiently solve P5. Since the total cost is the sum of individual user costs, selecting the user with the current lowest cost ensures local optimality at each step, ultimately achieving the global minimum cost. This approach (HFDA) leads to Algorithm 2.

The HFDA algorithm is divided into two modules: using Newton’s method to compute the minimization of $R_{i}^{n}\left(t_{i}^{n}\right)$ for each user *i*, and performing greedy selection through sorting to achieve the global minimum cost.
**Algorithm 2** HFDA (model dimension-aware non-convex low-cost scheduling algorithm).**Require:** $C_{e}^{n}$, user_local_gradient, user_h_dict, user_dataset_size_dict, $T_{\max}$, $M_{e}$, *V*, *B*, $N_{0}$, $p_{\max}$, $g_{\min}$, $g_{\max}$, α, γ, $\tau_{l}$, η, β1:*// calculate $S_{i}$*2:**for** each user $i\in C_{e}^{n}$ **do**3:   Calculate $S_{i}=S_{\mathrm{bottom}}+S_{\mathrm{top}}$ (Top-*k* gradients), s.t. $S_{\min}\leq S_{i}\leq S_{\max}$4:**end for**5:Calculate $\theta_{i}^{n}=\left(\delta_{i}^{n}/\beta \right)\times \left[{\left(1+\eta \beta \right)}^{\tau_{l}}-1\right]$ where $\delta_{i}^{n}=\delta_{i}^{n}\times \left(S_{i}/S_{\mathrm{full}}\right)$6:Initialize $\gamma =[M_{e}\times \left(1/\theta_{i}\right)/\sum_{j}\left(1/\theta_{j}\right)]$, z=γ, u=07:*// ADMM*8:**for** iter=1 **to** admm_max_iter **do**9:   Project γ to [(0.2,0.4)∪(0.6,0.8)]10:   Update z=γ+u, u=u+ρ×(γ−z)11:   **if** convergence **then**12:     **break**13:   **end if**14:**end for**15:Set $\Gamma_{i,n}=\gamma_{i}$, initialize $S_{1}^{n}=S_{2}^{n}=\ldots =S_{E}^{n}=\emptyset$16:*// User selection*17:**for** each edge server *e* **do**18:   **while** $|S_{e}^{n}|<M_{e}$ **and** $|C_{e}^{n}|>0$ **do**19:     **for** each user $i\in C_{e}^{n}$ **do**20:        Call Algorithm 1 with $S_{i}$ to get $R_{i}^{n}\left(t_{i}^{n,*}\right)$21:     **end for**22:     Select $i^{*}=\arg\min_{i\in C_{e}^{n}}R_{i}^{n}\left(t_{i}^{n,*}\right)$23:     Update $S_{e}^{n}=S_{e}^{n}\cup \left\{i^{*}\right\}$, $C_{e}^{n}=C_{e}^{n}\setminus \left\{i^{*}\right\}$24:   **end while**25:**end for**26:*// calculate power and frequency*27:**for** each selected user $i\in S_{e}^{n}$ **do**28:   $p_{i}^{n}=\max\left(p_{\min},\min\left(p_{\max},\frac{N_{0}\left(2^{S_{i}/\left(Bt_{i}^{n}\right)}-1\right)}{{\left(h_{i}^{n}\right)}^{2}}\right)\right)$29:   $g_{i}^{n}=\mathrm{clip}\left(\frac{\Gamma_{i,n}\tau_{l}\left|D_{i}\right|}{T_{\max}-t_{i}^{n}},g_{\min},g_{\max}\right)$30:**end for**31:*// update user queue state*32:**for** each user *i* **do**33:   $Q_{i}\left(n+1\right)=\max\left(Q_{i}\left(n\right)-a_{i}^{n}+\Gamma_{i,n},0\right)$ where $a_{i}^{n}=1$ if $i\in S_{e}^{n}$, else $a_{i}^{n}=0$34:**end for****Ensure:** $S_{1}^{n},S_{2}^{n},\ldots ,S_{E}^{n}$, $p^{n}$, $g^{n}$, user_$S_{i}$

## 4. Simulation

Two classic classification tasks in deep learning are employed to validate our proposed algorithm, involving two datasets: CIFAR-10 and Caltech-101.

The CIFAR-10 (Canadian Institute for Advanced Research 10) dataset comprises 10 mutually exclusive categories (airplane, automobile, bird, cat, deer, dog, frog, horse, ship, truck), with a total of 60,000 color RGB images of 32 × 32 pixels. Each category contains 6000 samples, including 50,000 samples for the training set and 10,000 samples for the test set. For the CIFAR-10 dataset, a simple convolutional neural network (CNN) is designed, and its architecture details are presented in Table 2.

The Caltech-101 dataset comprises 101 object categories and 1 background category, with a total of approximately 9144 images. The number of samples per category ranges from 40 to 800 (most categories have around 50 samples), and the image resolution is concentrated around 300 × 200 pixels, undergoing preprocessing such as size standardization and background removal.

For the Caltech-101 dataset, we also design a simple CNN adopting an architecture of “2 convolutional layers + 2 max-pooling layers + 1 adaptive average pooling layer + 3 fully connected layers”. The input is a 224 × 224 × 3 RGB image. First, it is processed by Conv1 (5 × 5 convolution kernel, 64 output channels, padding = 2, stride = 1) to obtain a 224 × 224 × 64 feature map, which is then downsampled to 111 × 111 × 64 via 3 × 3 max-pooling (stride = 2). Next, Conv2 (5 × 5 convolution kernel, 64 output channels, padding = 2, stride = 1) is applied to maintain the 111 × 111 × 64 feature map size, followed by 3 × 3 max-pooling (stride = 2) to generate a 55 × 55 × 64 feature map. After being fixed to 7 × 7 × 64 via adaptive average pooling and flattened into a 3,136-dimensional vector, the vector is sequentially processed by fully connected layers FC1 (384 outputs) and FC2 (192 outputs). Finally, FC3 outputs a 101-dimensional vector corresponding to the 101 categories. All convolutional layers and fully connected layers (except the output layer) use the ReLU activation function.

The parameter configurations for the remaining simulation experiments are specified in Table 3.

Subsequently, a specific scenario is constructed to apply our proposed algorithm: a square area with a size of 1000×1000 is divided into 16 uniform sub-regions, where 40 mobile users are randomly placed with random moving directions (adopting the random mobility model, which features mathematical tractability to facilitate theoretical derivation and performance evaluation). Although fixed-path models are more consistent with specific scenarios (e.g., unmanned aerial vehicles, UAVs), the random mobility model can cover diverse potential mobility patterns through multiple experiments, enabling a more comprehensive performance assessment. Notably, this model allows for evaluating the robustness of the algorithm under worst-case scenarios: if the algorithm maintains favorable performance under random mobility (characterized by frequent association changes and channel fluctuations), it is guaranteed to exhibit superior or comparable performance under other mobility patterns (e.g., fixed paths). Four edge servers are deployed in the scenario. Considering the impacts of server performance and bandwidth, a maximum number of selectable users is set for each edge server, with different configurations adopted for different datasets [9]. For the CIFAR-10 dataset, since it has fewer categories and the parameters of each user exert a greater impact, the maximum number of selectable users per edge server is set to 4. For the Caltech-101 dataset, due to its large number of categories (leading to potential differences in focus among different users), the maximum number of selectable users per edge server is set to 10.

Parameters of the model’s bottom layers (e.g., feature extraction layers of CNN, which can be replaced with another neural network) exhibit high sharing among users, contributing over 60% to global updates [34]; in contrast, parameters of the model’s top layers (e.g., classification heads) are highly personalized, and only 10–20% of key gradients (top 20% in absolute value) determine the update direction [35]. Therefore, a hierarchical partial upload strategy is adopted: the model is divided into bottom layers (convolutional layers) and top layers (fully connected layers); all parameters of the bottom feature layers are uploaded, while gradients of the top layers are filtered based on importance—specifically, gradients of the top layers are flattened and sorted by absolute value [36], and the top 30% of key gradients are selected for upload (gradient importance generally follows a long-tailed distribution, where the top-30% gradients can retain approximately 85–90% of gradient information while reducing the communication data volume by about 50%; although a fixed $k_{\mathrm{ratio}}=0.3$ is adopted in this paper, the algorithm design supports adaptive adjustment, which can be dynamically tuned according to gradient sparsity, training stages, or model convergence status to further improve the algorithm performance). The final upload dimension $S_{i}$ is calculated as $S_{i}=S_{\mathrm{bottom}}+S_{\mathrm{top}}$, which is constrained within the range $\left[S_{\min},S_{\mathrm{full}}\right]$. Here, $S_{\max}$ denotes the size of the full model ($S_{\mathrm{full}}$), and $S_{\min}$ represents the minimum upload dimension (for the Caltech-101 dataset, $S_{\min}$ is set to at least 70% of $S_{\mathrm{full}}$ to ensure data quality).

To reduce computational overhead and better simulate the diversity of each user’s dataset, different sharding processes are performed for each dataset, and the sharded data are randomly assigned to each user while ensuring that all users have datasets of the same size. Detailed information is given in Table 4.

### 4.1. Performance of HFDA

We compare the proposed HFDA algorithm with the following baseline algorithms in terms of accuracy and energy consumption.

#### 4.1.1. Baseline 1: Random Scheduling (RS) Algorithm

The RS algorithm adopts random user selection. Each edge server randomly selects $M_{e}$ users from the associated users to participate in training, but uses Algorithm 1 (convex optimization) to perform resource allocation for the selected users. The optimal communication time, transmission power, and CPU frequency are solved by the Newton method to minimize the energy consumption per round.

#### 4.1.2. Baseline 2: Random Allocation (RA) Algorithm

The RA algorithm adopts a completely random strategy. It not only randomly selects users but also randomly allocates resources (transmission power and CPU frequency) within the feasible range. It does not consider channel quality, data quality, or energy consumption, nor does it consider time constraints and energy consumption optimization, and performs no optimization.

#### 4.1.3. Baseline 3: Loss-Driven (LD) Algorithm

The LD algorithm performs user selection based on the local training loss. It gives priority to users with small local training loss (these users have a smaller difference between their local model and the global model, and contribute more to model updates), and then uses Algorithm 1 to optimize resource allocation for the selected users.

#### 4.1.4. Baseline 4: Constant Participation Rate (CP) Algorithm

The CP algorithm adopts a fixed participation rate strategy. Each edge server randomly selects users according to a fixed participation rate, and then uses Algorithm 1 to optimize resource allocation for the selected users.

#### 4.1.5. Baseline 5: Hierarchical Federated Edge Learning Algorithm (HFEEL)

The HFEEL algorithm adopts a joint optimization strategy of single-round energy consumption and gradient divergence. It calculates a comprehensive selection index based on users’ gradient divergence ($\delta_{i}^{n}$, the smaller the value, the better the gradient consistency) and single-round energy consumption, and gives priority to users with small gradient divergence and low energy consumption. Then, it uses Algorithm 1 to perform resource allocation optimization [25].

#### 4.1.6. Baseline 6: Low-Cost Scheduling Algorithm (LCSA)

The LCSA adopts a long-term energy consumption constraint and dynamic participation rate optimization strategy [18]. It realizes long-term fairness guarantee and energy consumption constraint through a virtual queue mechanism ($Q_{i}\left(n\right)$). It dynamically calculates the participation rate ($\Gamma_{i,n}=M_{e}\times \left(1/\theta_{i}^{n}\right)/\sum \left(1/\theta_{j}^{n}\right)$) according to users’ gradient divergence ($\theta_{i}^{n}$, calculated by $\delta_{i}^{n}$), rather than using a fixed value. In terms of user selection, the LCSA gives priority to users with low cost. The cost function is $R_{i}^{n}=V\times \left(E_{\mathrm{calculate}}+E_{\mathrm{transmit}}\right)-Q_{i}$, which comprehensively considers single-round energy consumption (communication energy consumption and computing energy consumption) and virtual queue value to achieve long-term energy consumption–performance trade-off. In terms of resource allocation, the LCSA uses Algorithm 1 (convex optimization method, solved by the Newton method) to calculate the optimal communication time, transmission power, and CPU frequency for each user.

### 4.2. Results

**Figure 3:**
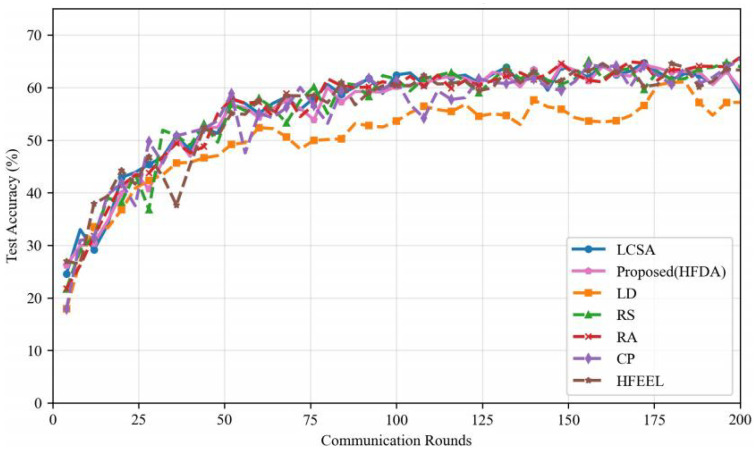
Performance comparison of different algorithms—CIFAR-10 (accuracy).

**Figure 4:**
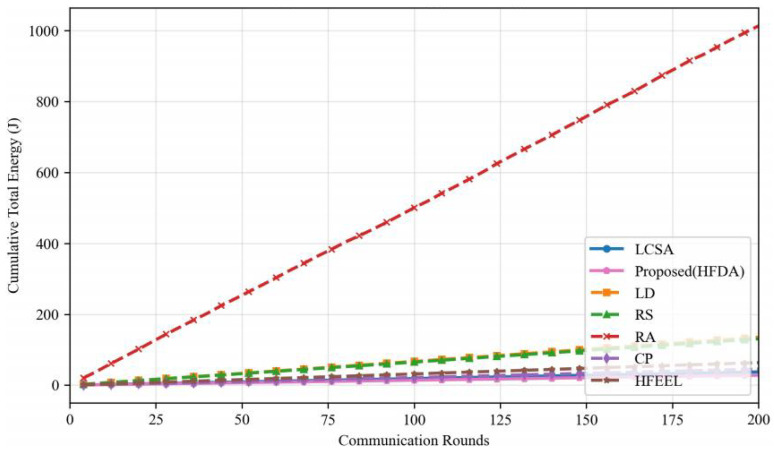
Performance comparison of different algorithms—CIFAR-10 (energy consumption).

**Figure 5:**
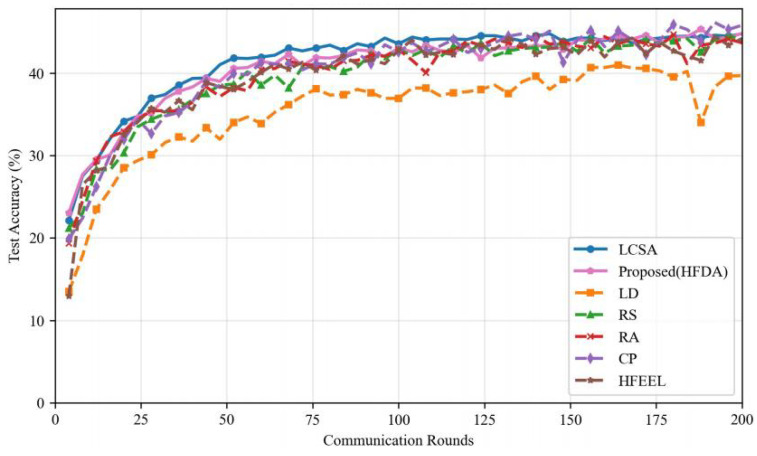
Performance comparison of different algorithms—Caltech-101 (accuracy).

**Figure 6:**
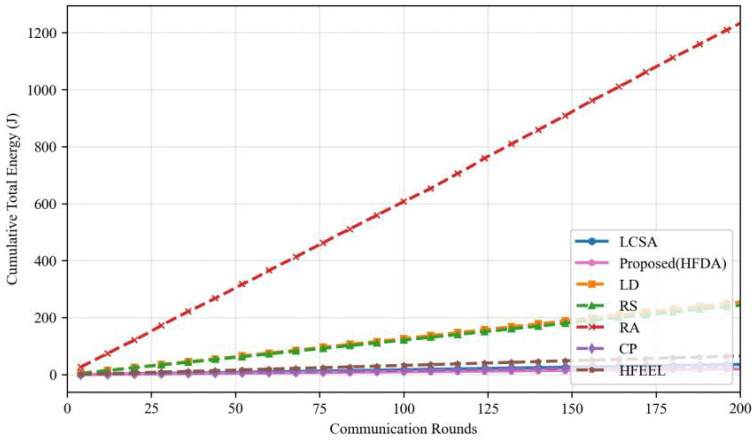
Performance comparison of different algorithms—Caltech-101 (energy consumption).

As shown in Figure 3, Figure 4, Figure 5 and Figure 6, we present the comparison of test accuracy and energy consumption between our proposed HDFA algorithm and other resource allocation algorithms on the CIFAR-10 and CELTECH-101 datasets. For the above two datasets, our algorithm achieves a balance between accuracy and energy consumption, maximizing the initial optimization goal of minimizing energy consumption while ensuring high accuracy.

**Figure 7:**
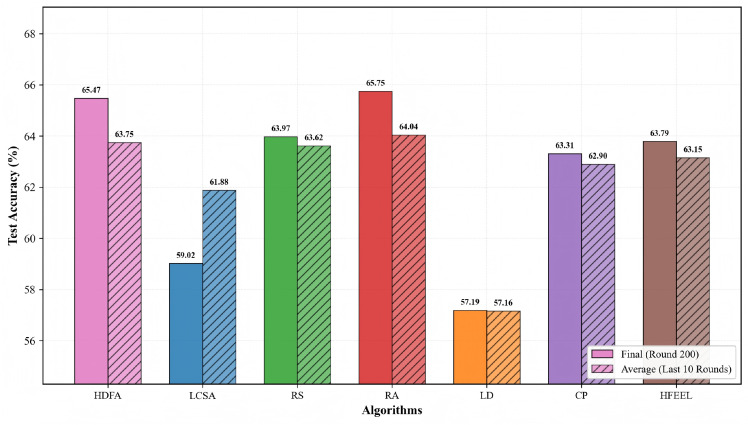
Test accuracy comparison—CIFAR-10.

**Figure 8:**
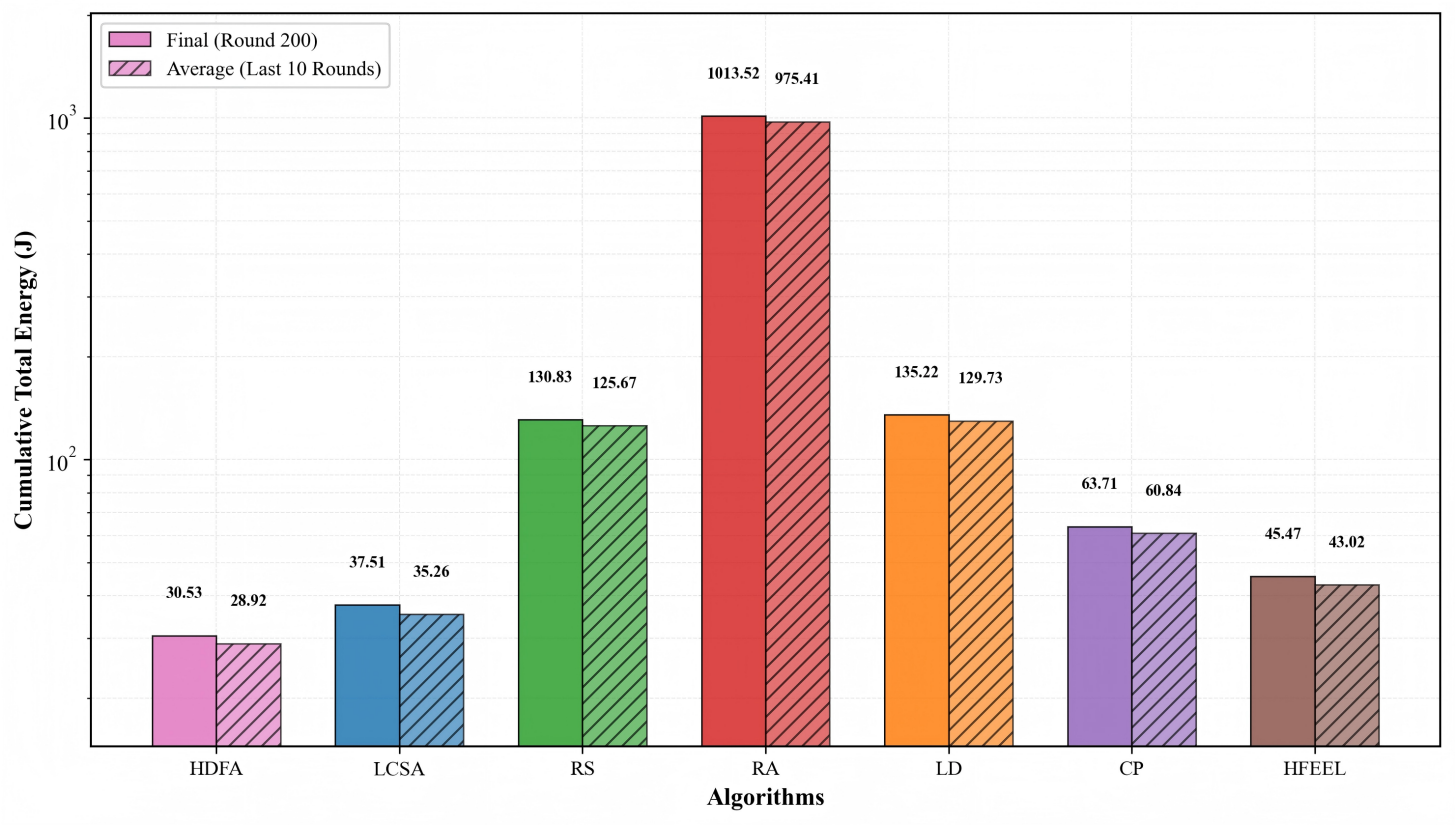
Energy consumption comparison—CIFAR-10.

On the CIFAR-10 dataset, the performance of our algorithm in terms of accuracy and energy consumption is shown in the following figures:

Figure 7 and Figure 8 illustrate the accuracy and energy consumption performance of various algorithms on the CIFAR-10 dataset after 200 training rounds. Figure 7 shows that HDFA achieves a final accuracy of 65.47% and an average accuracy of 63.75%. In terms of accuracy, HDFA is second only to RA (65.75%) and significantly outperforms LCSA (59.02%) and LD (57.19%). Figure 8 indicates that HDFA has a cumulative total energy consumption of 30.53 J (with an average of 28.92 J), which is the lowest among all algorithms; the cumulative total energy consumption of LCSA is 37.51 J (average 35.26 J), HFEEL is 45.47 J (average 43.02 J), CP is 63.71 J (average 60.84 J), RS and LD are 130.83 J and 135.22 J, respectively, and RA reaches as high as 1013.5 J. The results demonstrate that HDFA achieves the lowest energy consumption while maintaining high accuracy, reflecting an excellent energy consumption–performance trade-off.

On the CIFAR-10 dataset, the energy consumption improvement of our algorithm compared with other resource allocation algorithms is significant, and the improvement effect is shown in the following figures.

**Figure 9:**
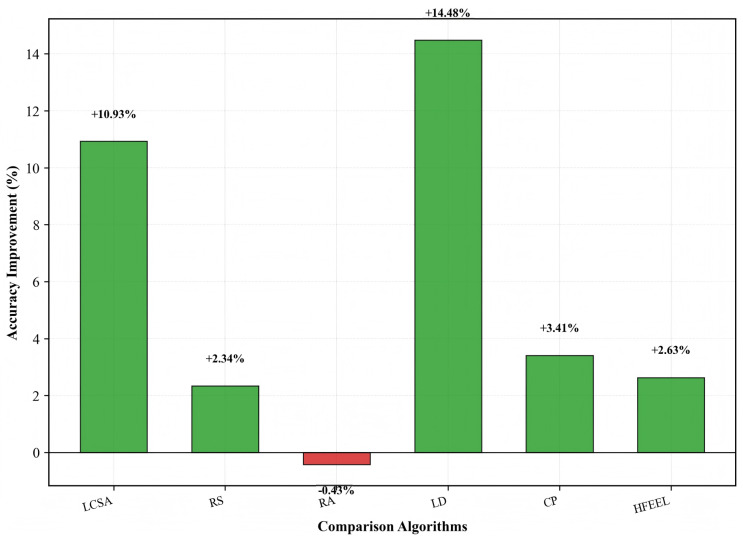
Accuracy improvement of HDFA compared to baseline algorithms—CIFAR-10.

**Figure 10:**
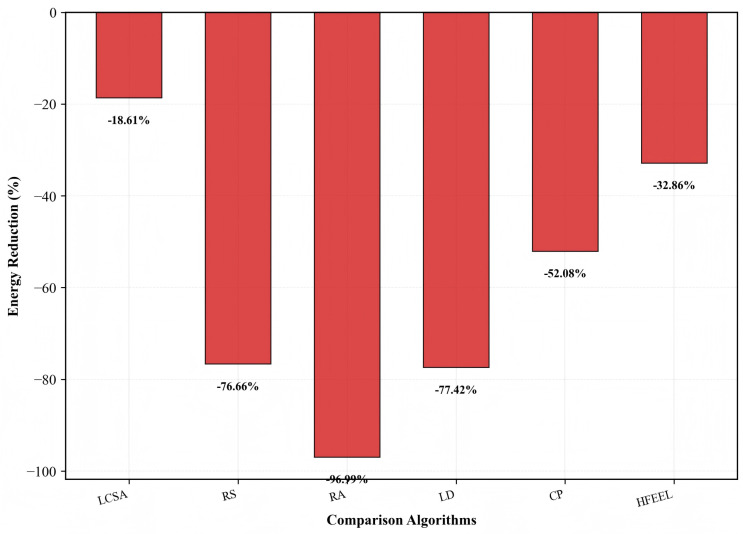
Energy reduction of HDFA compared to baseline algorithms—CIFAR-10.

**Figure 11:**
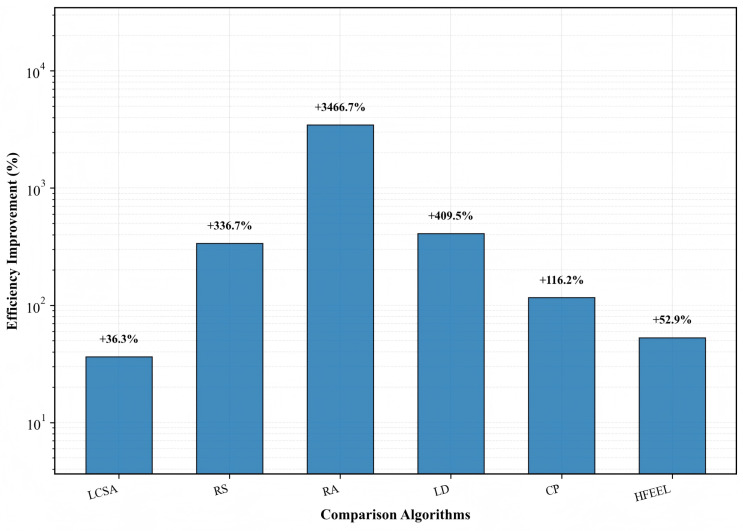
Improvement in accuracy–energy balance performance of HDFA compared to baseline algorithms—CIFAR-10.

Figure 9, Figure 10 and Figure 11 demonstrate the performance improvement of HDFA relative to each baseline algorithm. Figure 9 shows that HDFA improves accuracy by 14.48%, 10.93%, 3.41%, 2.63%, and 2.34% compared with LD, LCSA, CP, HFEEL, and RS, respectively, and only decreases by 0.43% compared with RA. Figure 10 indicates that HDFA reduces energy consumption by 96.99%, 77.42%, 76.66%, 52.08%, 32.86%, and 18.61% relative to RA, LD, RS, CP, HFEEL, and LCSA, respectively. Figure 11 shows that HDFA achieves significant improvement in accuracy–energy balance performance: it improves accuracy–energy balance performance by 3466.7%, 409.5%, 336.7%, 116.2%, 52.9%, and 36.3% compared with RA, LD, RS, CP, HFEEL, and LCSA, respectively. Overall, HDFA is comparable to the optimal baseline (RA) in terms of accuracy, while significantly reducing energy consumption and achieving a remarkable improvement in accuracy–energy balance performance. This verifies the effectiveness of the partial-dimensional model upload and non-convex participation rate optimization strategies.

On the Caltech-101 dataset, the accuracy and energy consumption performance of our algorithm is shown in the following figures.

**Figure 12:**
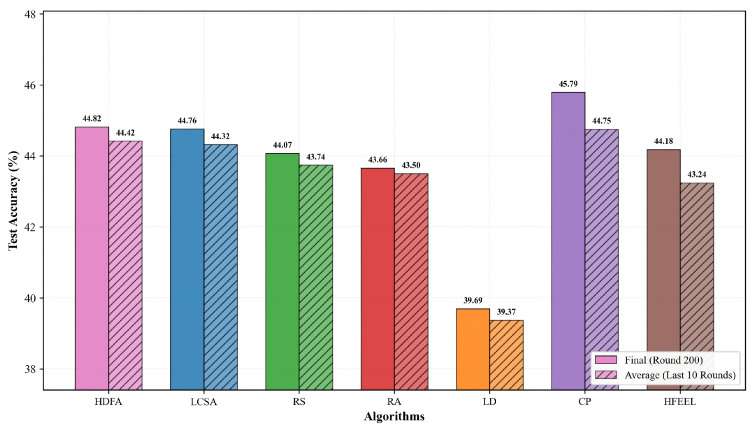
Test accuracy comparison—Caltech-101.

**Figure 13:**
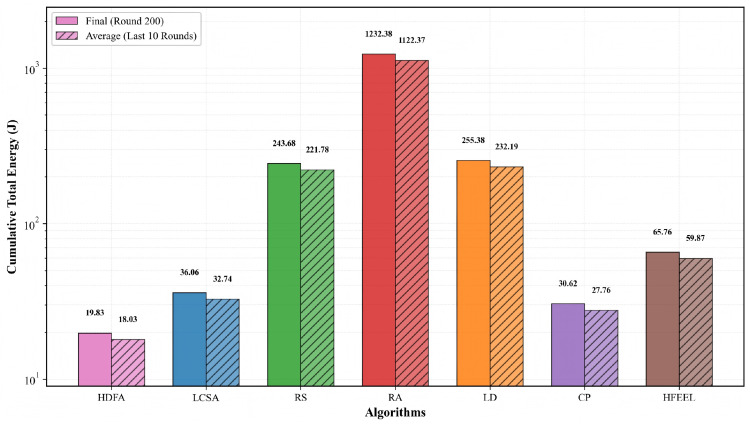
Energy consumption comparison—Caltech-101.

Figure 12 and Figure 13 present the comparison of accuracy and energy consumption of different algorithms on the Caltech-101 dataset. Figure 12 shows that the final accuracies of HDFA and LCSA are 44.82% and 44.76%, respectively, with average accuracies of 44.42% and 44.32%—both comparable to those of other algorithms. CP achieves the highest accuracy (45.79%), LD the lowest (39.69%), and RS, RA, and HFEEL fall within the range of 43.66–44.18%. Figure 13 displays the cumulative total energy consumption (on a logarithmic scale): HDFA has the lowest consumption (19.83 J), followed by LCSA (36.06 J), CP (30.62 J), HFEEL (65.76 J), RS (243.68 J), LD (255.38 J), and RA (1232.38 J, the highest). The results show that HDFA maintains accuracy comparable to the baselines while achieving significantly lower energy consumption, embodying a favorable energy consumption–performance trade-off.

On the Caltech-101 dataset, the energy consumption improvement of our algorithm compared with other resource allocation algorithms remains significant, and the specific effect is shown in the following figures.

**Figure 14:**
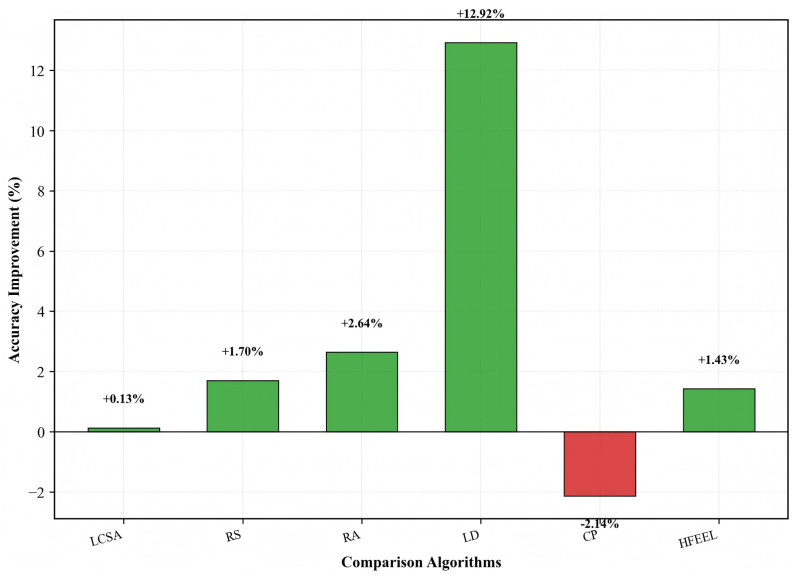
Accuracy improvement of HDFA compared to baseline algorithms—Caltech-101.

**Figure 15:**
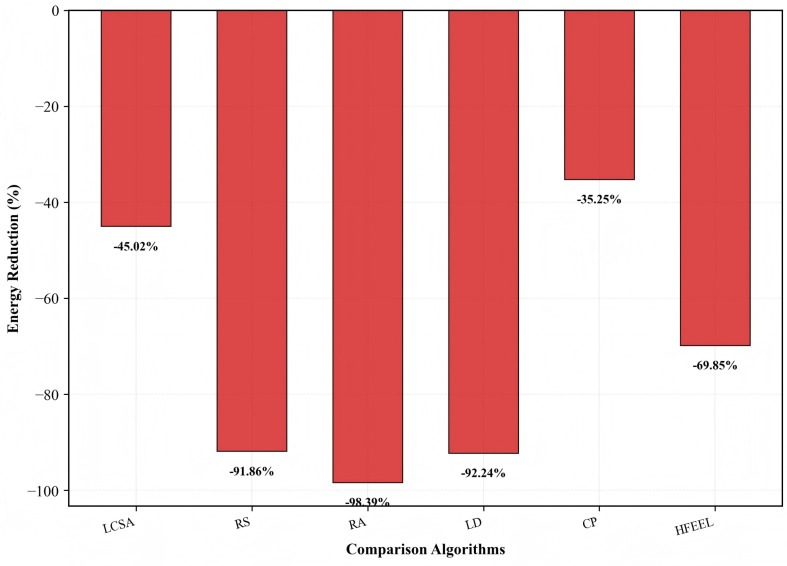
Energy reduction of HDFA compared to baseline algorithms—Caltech-101.

**Figure 16:**
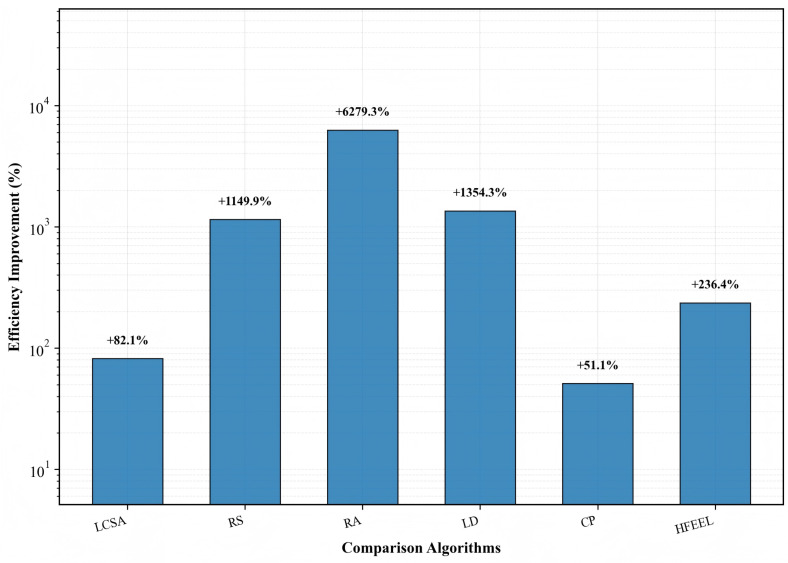
Improvement in accuracy–energy balance performance of HDFA compared to baseline algorithms—Caltech-101.

Figure 14, Figure 15 and Figure 16 exhibit the performance improvement of HDFA relative to each baseline algorithm. Figure 14 shows the accuracy improvement: HDFA increases accuracy by 12.92% compared with LD, 2.64%, 1.70%, and 1.43% compared with RA, RS, and HFEEL, respectively, 0.13% compared with LCSA, and decreases by 2.14% compared with CP. Figure 15 shows the energy consumption reduction: HDFA cuts energy consumption by 98.39%, 92.24%, and 91.86% relative to RA, LD, and RS, respectively, and by 69.85%, 45.02%, and 35.25% relative to HFEEL, LCSA, and CP, respectively. Figure 16 shows the accuracy–energy balance performance (on a logarithmic scale): HDFA enhances accuracy–energy balance performance by 6279.3%, 1354.3%, and 1149.9% compared with RA, LD, and RS, respectively, and by 236.4%, 82.1%, and 51.1% compared with HFEEL, LCSA, and CP, respectively. Overall, HDFA is comparable to or better than most baselines in accuracy, while significantly reducing energy consumption and achieving notable accuracy–energy balance performance—this verifies its effectiveness on complex datasets.

Since the selection strategy of the RA algorithm is completely random in both user selection and resource allocation, it achieves the worst energy consumption under similar FL performance. The LD and RS algorithms only consider optimization based on a single condition; although they show significant improvements over RA, their overall performance remains unsatisfactory. The HFEEL algorithm takes more comprehensive factors into account in the short term, thus achieving further performance improvement, but there is still a performance gap compared with the CP, LCSA, and HDFA algorithms. We focus more on the CP, LCSA, and HDFA algorithms, which perform well in both accuracy and energy consumption.

Although the accuracy of our HDFA algorithm is slightly lower than that of LCSA, it ranks second overall—and this is logically reasonable. The reason is that our algorithm does not upload the full-dimensional model of users, which leads to a slight deviation compared with LCSA (which uploads full-dimensional models). However, experimental results show that the impact of these deviations is minimal and acceptable.

A very small reduction in accuracy is accompanied by a substantial decrease in energy consumption. Our algorithm achieves the minimization of energy consumption on both datasets. While its accuracy is only slightly inferior to that of LCSA, our algorithm further reduces communication energy consumption. For the more complex classification task on the Caltech-101 dataset, the energy consumption of LCSA is slightly worse than that of CP (which focuses solely on low energy consumption). In contrast, our algorithm not only ensures accuracy comparable to LCSA but also achieves lower total energy consumption than CP, thus delivering better performance. This fully confirms our hypothesis: by uploading core model parameters instead of full-dimensional models, communication energy consumption is reduced at the source. This approach not only does not have a significant impact on model accuracy but also further reduces total energy consumption, ultimately achieving better performance.

The ’accuracy–energy balance performance’ mentioned in the paper is quantitatively based on the ratio of model test accuracy to total energy consumption (Acc/Etotal), which is used to intuitively demonstrate the comprehensive advantage of the algorithm in reducing energy consumption while maintaining accuracy. The core performance evaluation still relies on total energy consumption and test accuracy.

### 4.3. Computational Complexity and Large-Scale Scalability Analysis of HFDA

The HFDA algorithm integrates four optimization methods: Lyapunov optimization, Alternating Direction Method of Multipliers (ADMM), Newton’s iteration, and greedy selection. Notably, each module achieves linear or near-linear complexity, ensuring the algorithm’s excellent scalability. Specifically,

- **Lyapunov Optimization**: It implements long-term energy consumption constraints through a virtual queue mechanism, with a complexity of O(U) (where *U* denotes the total number of terminals). For each terminal, only one addition operation and one maximum value operation are required, resulting in negligible computational overhead.
- **Newton’s Iteration**: This method solves the single-user resource allocation optimization problem to find the optimal communication time through iterative updates. Its complexity is O(U×I), where *I* is the number of iterations (averaging 8 times and up to 50 times). The resource allocation problem for each terminal is solved independently, involving first-order and second-order derivative calculations, with approximately 60 floating-point operations per iteration.
- **ADMM Algorithm**: It addresses the non-convex participation rate constraint $\Gamma_{i}\in \left[\left(0.2,0.4\right)\cup \left(0.6,0.8\right)\right]$ by alternately updating primal variables, dual variables, and Lagrange multipliers. The complexity is $O(E\times I_{\mathrm{admm}}\times U_{e})$, where *E* is the number of edge servers (fixed at 4), $I_{\mathrm{admm}}$ is the number of ADMM iterations (averaging 8 times and up to 20 times), and $U_{e}$ is the average number of terminals per edge server (U/E). Each iteration involves updating the participation rate, projection, and dual variables, with a total of approximately 64U operations.
- **Greedy Selection Algorithm**: It selects users by sorting their costs, with a complexity of $O(E\times U_{e}\times \log U_{e})$. The sorting operation constitutes the main computational overhead.

Thus, the overall complexity of HFDA is $O(U\times I+E\times U_{e}\times \log U_{e})$, where the dominant term is O(U×I). Since *I* is a constant (approximately 8), the computational load grows linearly with the number of terminals without explosive growth. Theoretical analysis indicates that even when scaled to 10,000 terminals, the single-round computation time is less than 5 ms, and the total computation time for 500 training rounds is less than 2.5 s—far shorter than the actual training time (typically several hours)—ensuring no performance bottlenecks.

HFDA exhibits excellent performance in large-scale scenarios. Based on complexity analysis, the computation time grows linearly with the number of terminals for different scales:

- Less than 0.05 ms per round for 100 terminals;
- Less than 0.5 ms per round for 1000 terminals;
- Less than 5 ms per round for 10,000 terminals;
- Less than 50 ms per round for 100,000 terminals (parallelizable to 6.25 ms).

More importantly, the algorithm is designed to support parallelization: (1) In Newton’s iteration, the resource allocation problem for each terminal is completely independent and can be computed in parallel. (2) In greedy selection, the user selection process of each edge server is mutually independent and can be executed in parallel. Using an eight-core CPU can reduce the computation time by eight times, further enhancing scalability. Additionally, experimental results based on the CIFAR-10 dataset show that HFDA saves 6.7 J of energy (40.7%) within 13 training rounds. Theoretical analysis indicates that for the actual experimental scale (200 terminals), the estimated computation overhead is less than 2% (negligible); even when scaled to 1000 terminals, the overhead proportion remains less than 10% (still acceptable); only in ultra-large-scale scenarios (10,000 terminals) does the computation overhead approach the energy savings from training, but it can be further reduced through parallelization optimization. Therefore, the algorithm maintains excellent performance in large-scale scenarios, and the computational load will not offset the energy-saving effect.

## 5. Conclusions

In this paper, we presented HFDA, a rigorously designed HFL framework that halves uplink energy without sacrificing accuracy by uploading only the critical model subspace. Using a Lyapunov-guided, ADMM-solved non-convex formulation, HFDA jointly optimizes dimension selection masks and user participation while guaranteeing convergence. Extensive experiments on CIFAR-10 and Caltech-101 show identical accuracy to the best competitor (LCSA) and the lowest cumulative energy among all baselines, confirming that the proposed “critical-dimension upload + dynamic scheduling” strategy is immediately deployable for IoT and MEC scenarios where every joule and bit count.

## Figures and Tables

**Table 1:** Notations and their descriptions.

Notation	Descriptions
*B*	Index set of mobile users
E	Index set of edge servers
$d_{i}^{m}$	Direction of user *i*
*s*	Speed of mobile users
$w_{i}^{\tau ,n}$	Local model of user *i* at *n*-th communication round and τ-th local epoch
$w_{e}^{n}$	Edge model of edge server *e* at *n*-th communication round
$w^{n}$	Global model at *n*-th communication round
$f_{i}\left(\cdot \right)$	Local loss function of user *i*
$F_{e}^{n}\left(\cdot \right)$	Edge loss function of edge server *e* at *n*-th communication round
F(·)	Global loss function
$v_{e}^{n}$	Auxiliary edge model of edge server *e* at *n*-th communication round
$v^{n}$	Auxiliary global model of central server at *n*-th communication round
$u_{e}^{\tau ,n}$	Edge model of edge server *e* at *n*-th round and τ-th local epoch
$u^{\tau ,n}$	Global model at *n*-th communication round and τ-th local epoch
$D_{i}$	Dataset of user *i*
$|D_{i}|$	Data size of dataset $D_{i}$
a	User selected indicator
g	Computing frequency vector
p	Transmit power vector
*C*	Bandwidth of channels
$\Gamma_{i}^{n}$	Attendance rate of user *i* at *n*-th communication round
$M_{e}$	Channel number of BS *e*
$h_{i}^{n}$	Channel gain of user *i* at *n*-th communication round
$S_{i}$	Model upload size of user *i*
$\tau_{e}$	Edge aggregation number between two global aggregation
$\tau_{l}$	Local epoch number
$C_{e}^{n}$	User set of edge server *e* at *n*-th communication round
$T^{\max}$	Communication round time
$\delta_{i}^{n}$	Upper bound of the distance between $f_{i}\left(w\right)$ and $F_{e}^{n}\left(w\right)$
$\Delta_{e}^{n}$	Upper bound of the distance between $F_{e}^{n}\left(w\right)$ and F(w)
$\delta^{n}$	The weighted average of $\delta_{i}^{n}$
$\Delta^{n}$	The weighted average of $\Delta_{e}^{n}$
$S_{\mathrm{full}}$	Full-dimensional size of uploaded model
ρ	Model error rate

**Table 2:** Architecture details of the CNN for CIFAR-10.

Layer Type	Parameters
Input	-
Conv1	5 × 5 kernel, 64 channels, padding = 2, stride = 1
Max-Pooling1	3 × 3 kernel, stride = 2
Conv2	5 × 5 kernel, 64 channels, padding = 2, stride = 1
Max-Pooling2	3 × 3 kernel, stride = 2
Flatten	-
FC1	3136→384
FC2	384→192
FC3	192→10

**Table 3:** Architecture details of the CNN for Caltech-101.

Layer Type	Parameters
Input	-
Conv1	5 × 5 kernel, 64 channels, padding = 2, stride = 1
Max-Pooling1	3 × 3 kernel, stride = 2
Conv2	5 × 5 kernel, 64 channels, padding = 2, stride = 1
Max-Pooling2	3 × 3 kernel, stride = 2
Adaptive Avg Pooling	Fixed to 7 × 7 × 64
Flatten	-
FC1	3136→384
FC2	384→192
FC3	192→101

**Table 4:** Dataset sharding and configuration parameters.

Parameter Item	CIFAR-10	Caltech-101
Number of Dataset Categories	10	101
Training Set Size	50,000	8000–9000
Number of Shards per Category	8	8
Number of Shards per User	2	20
Total Number of Base Shards	80	808

## Data Availability

The original contributions presented in this study are included in the article. Further inquiries can be directed to the corresponding author.
